# Brain health care accelerator: A primary care–centered model for scaling dementia diagnosis and care

**DOI:** 10.1002/alz.71405

**Published:** 2026-04-16

**Authors:** Jeffrey M. Burns, Jill K. Morris, Eric D. Vidoni, Ryan A. Townley, Lindsey R. Gillen, Timothy R. Smith, Janell E. Jones, Melody B. Nelson, Dinesh Pal Mudaranthakam, Jaime Perales‐Puchalt, Tina Lewandowski, C. Michelle Niedens, Adam C. Parks, Amanda M. Brunette, Andrew J. Aschenbrenner, Taylor I. Bucy, Rachel C. Forcino, Jennifer L. Woodward

**Affiliations:** ^1^ Department of Neurology University of Kansas Medical Center Kansas City Kansas USA; ^2^ University of Kansas Alzheimer's Disease Research Center, University of Kansas Medical Center Kansas City Kansas USA; ^3^ University of Kansas Health System Kansas City Kansas USA; ^4^ Department of General Pediatrics University of Kansas Medical Center Kansas City Kansas USA; ^5^ Department of Family Medicine University of Kansas Medical Center Kansas City Kansas USA; ^6^ Department of Pathology and Laboratory Medicine University of Kansas Medical Center Kansas City Kansas USA; ^7^ Department of Biostatistics & Data Science University of Kansas Medical Center Kansas City Kansas USA; ^8^ Department of Population Health University of Kansas Medical Center Kansas City Kansas USA

**Keywords:** Alzheimer disease, Biomarkers, Blood, Delivery of Health Care, Early Diagnosis, Integrated, Primary Health Care

## Abstract

Plasma biomarkers and disease‐modifying therapies for early Alzheimer's disease have created an urgent need for scalable care models. In this *Perspectives*, we describe how a health system is adapting dementia care delivery through a primary care provider (PCP)–centered initiative designed to improve diagnostic readiness and care capacity. The program integrates (1) blood‐based biomarker testing into clinical workflows, (2) a Cognitive Assessment Visit (CAV) supported by Electronic Health Record–embedded decision tools, and (3) memory care co‐management. Early implementation demonstrates that PCPs can be empowered to diagnose and manage cognitive impairment through structured workflows, targeted training, and specialist collaboration. In the first 26 weeks, 1,331 plasma p‐tau217 tests were ordered by 104 clinicians, including 58 PCPs; 59 CAVs were completed by nine PCPs; and more than 330 asynchronous e‐consults expanded specialist access. These results highlight the feasibility and challenges of integrating biomarker‐informed dementia care within primary care workflows.

## Highlights

- Primary care‐centered practice models can enable scalable dementia evaluation.
- Standardized pathways support rapid, scalable Alzheimer's biomarker use across clinics.
- Co‐management structures expand specialist access and improve coordinated dementia care without increasing specialty clinic burden.

## 1 INTRODUCTION

Advances in Alzheimer's disease (AD) diagnostics and therapeutics make earlier, more accurate diagnoses possible. Blood‐based biomarkers allow minimally invasive detection of AD pathology,^1^, ^2^, ^3^ and anti‐amyloid therapies provide benefit when started early.^4^ The rising prevalence of AD and related dementias^2^ coupled with shortages of dementia specialists,^5^, ^6^, ^7^, ^8^ exposes the inability of current systems to meet present and future needs.^6^, ^7^, ^9^ These tools will thus not reach their potential without new models of care that deliver them at a scale to meet the growing need. In this *Perspectives*, we call for changes in how dementia care is delivered and describe one large health system's pragmatic initial efforts to scaling diagnosis and care pathways.

Importantly, the opportunity for early intervention typically occurs when patients are still predominately managed in primary care settings. Primary care providers (PCPs) report limited training, time, and workflows, leading to delayed or missed diagnoses.^10^, ^11^, ^12^ PCPs need to be equipped to diagnose, manage, and coordinate effectively with specialty‐trained providers.^13^ National guidelines emphasize embedding cognitive assessments, biomarkers, and electronic health record (EHR) decision support into primary care.^13^ Similar strategies have transformed diabetes and hypertension care, where structured workflows and team‐based approaches improved detection and treatment.^14^, ^15^, ^16^ Comprehensive dementia care requires a comparable transformation to redefine primary care as the hub for early detection and management while preserving specialist capacity for complex cases.^17^ Innovation towards this goal is progressing rapidly including the development of new staffing roles,^18^ and accelerated evaluation models that leverage blood‐based AD biomarkers and digital evaluation tools.^19^

Against this backdrop of innovation, we developed the Brain Health Care Accelerator (BHCA), a health system–wide, primary care–centered model with a goal of integrating and operationalizing approaches to enable earlier and more accurate dementia diagnosis and management. The BHCA leverages plasma AD biomarkers (i.e., phosphorylated tau 217 [p‐tau217], structured EHR workflows, and memory care co‐management, and is supported by strategies and programs that promote adoption and sustainability. Here, we describe the BHCA's design, early implementation, and preliminary measures of adoption and utility.

### 1.1 Program Description: The Brain Health Care Accelerator (BHCA)

The KU Memory Care Clinic (established in 2004) is closely integrated with the KU Alzheimer's Disease Research Center (KU ADRC), with four academic neurologists and six advanced practice providers (APPs). The clinic and the KU ADRC's history of integrating specialty care, health system infrastructure, and research provided the foundation for the BHCA.

The BHCA did not emerge from a single, prospectively staged implementation plan. Instead, it developed in response to the recognition that our traditional memory clinic model could not keep pace with rising demand and a shifting landscape of new biomarkers and anti‐amyloid therapies. The program evolved through a pragmatic, iterative process led by a core group of clinician and researcher stakeholders and leveraged existing clinical programs, leadership structures, and health system infrastructure. To inform its early design we conducted formative interviews in 2024 with ten PCPs across Family Medicine, Internal Medicine, and Geriatrics. PCPs were enthusiastic about a larger role in dementia care but cited barriers, including limited training, time constraints, workflow issues, and limited access to specialists. They emphasized the need for concise training, EHR‐embedded tools, and clear referral and co‐management pathways with timely specialist feedback, echoing concerns noted in prior studies.^10^, ^11^, ^12^

**FIGURE 1:**
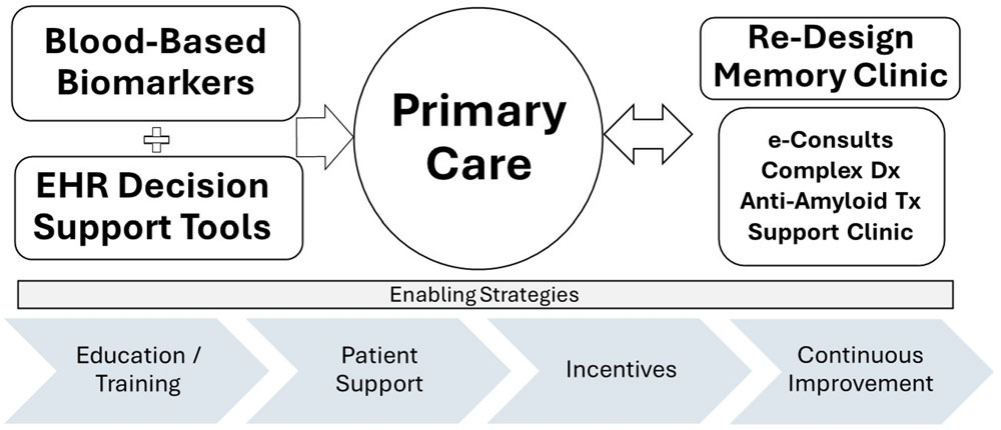
The Brain Health Care Accelerator (BHCA) integrates blood‐based biomarkers and electronic health record (EHR) decision support tools into primary care workflows to enable earlier and more accurate dementia diagnosis. Primary care serves as the hub, supported by redesigned memory clinic pathways that include e‐consults, complex diagnostic evaluations, anti‐amyloid treatment management, and comprehensive support clinics. Implementation and sustainability are reinforced by education and training, patient support, incentives, and emphasizing a culture of continuous improvement.

After establishing our needs, we organized the BHCA around three core components (Figure 1): (1) blood‐based biomarkers, (2) a Cognitive Assessment Visit (CAV) workflow with EHR‐embedded decision support, and (3) memory care co‐management clinics. These are supported by implementation strategies (training, incentives, clinical champions, and iterative refinement) and system‐enablers (caregiver support, lifestyle education, professional training programs, and new workflows) that further enhance feasibility, adoption, and sustainability.

Implementation of the BHCA occurred in parallel rather than sequentially, with biomarker integration, CAV development, and memory clinic reorganization informing one another through ongoing feedback. Barriers identified through PCP interviews and early clinical use were integrated into workflows, EHR tools, staffing models, specimen handling procedures, and referral pathways through rapid‐cycle refinement rather than concrete protocols. As a result, the BHCA reflects real‐world clinical constraints, including uneven adoption across the health system and the need for continued adaptation, rather than a fixed or fully standardized implementation package ready for direct replication.

BHCA was funded primarily through reorganization of existing clinical infrastructure and routine reimbursement (billable visits and services), rather than creation of a separately funded program. Startup acceleration came from the Davos Alzheimer's Collaborative Healthcare System Preparedness Accurate Diagnosis program that supported biomarker implementation and from the integrated NIH‐funded ADRC environment linked to clinical operations. The principal investment was leadership commitment to operational change in response to patient demand and access constraints.

To enhance interpretability, we retrospectively applied the RE‐AIM framework to organize reporting of early implementation processes and operational outcomes (Table 1), while longer‐term patient‐ and caregiver‐level outcomes are being evaluated. As we move towards scaling in our health system, we are employing the the Exploration, Preparation, Implementation and Sustainment (EPIS) framework to provide a practical roadmap for recognizing contextual factors critical to BHCA scale‐up.^20^ Through EPIS we have begun addressing inner contextual factors (engaging PCPs in tool development, aligning BHCA with existing workflows), outer contextual factors (CMS reimbursement, policy alignment), and bridging activities (cross‐functional teams, pilot testing, champion identification). For sustainment, BHCA components are embedded into routine workflows, and a dissemination package of training resources, EHR templates, and peer‐reviewed publications will be developed. This integrated RE‐AIM and EPIS approach is expected to enhance BHCA scalability for broad adoption and sustained use within our health system.

**TABLE 1:** Reach, effectiveness, adoption, implementation and maintenance (RE‐AIM) preliminary evaluation.

RE‐AIM domain	Operationalization	Data source	Early indicators
Reach	Patients eligible for and receiving BHCA components (biomarker testing, CAVs, e‐consults)	EHR, laboratory records	1,331 plasma p‐tau217 tests ordered; 59 CAVs completed in first 6 months
Adoption	Uptake by clinicians and clinical settings across the health system	EHR audit logs	104 clinicians ordered biomarkers; 58 were PCPs across Family and Internal Medicine
Implementation	Delivery of core intervention components and pragmatic workflow adaptations over time	Program records, EHR configuration history	Iterative refinement of CAV duration, SmartPhrases, referral workflows, and sub‐clinic triage
Effectiveness	Proximal system‐level outcomes reflecting diagnostic and care‐pathway changes	EHR	30% increase in MCI diagnoses in Family Medicine without increased memory clinic referrals

BHCA, Brain Health Care Accelerator; CAV, Cognitive Assessment Visit; CPT, Centers for Medicare and Medicaid Services Current Procedural Terminology EHR, electronic health record; MCI, mild cognitive impairment.

### 1.2 Blood‐Based Biomarkers

The KU ADRC initiated efforts to introduce plasma biomarkers into clinical practice in 2024. We took a pragmatic approach and embedded the testing directly into practice, with orders and results integrated into the EHR (Epic Systems Corp, Madison, WI) to minimize barriers and support point‐of‐care use. Formal, comprehensive training (biomarkers ordering, use in a clinic workflow, and interpretation for AD diagnosis/treatment) was delivered initially within the family medicine department, with targeted education also provided in Neurology. All providers in the health system had access to the biomarker test order and a brief online biomarker training video module,

The KU ADRC Biomarker Core, in collaboration with the KU Health System's CAP/CLIA‐certified laboratory, validated a laboratory‐developed test (LDT) for plasma ptau217 (Lumipulse G1200, Fujirebio Diagnostics, Inc.), which was deployed system‐wide in April 2025 with 24‐hour result turnaround allowing real‐world examination of workflow implications and use patterns. In‐house implementation reduced friction, shortened turnaround times, and enabled rapid‐cycle learning.

#### 1.2.1 Early biomarker uptake

In the first 26 weeks, 1,331 tests were ordered by 104 clinicians (70 physicians, 15 APPs, and 19 residents or fellows) across several departments. Providers in the Memory Care Clinic quickly incorporated p‐tau217 into the routine diagnostic workup, accounting for 975 orders (73.4% of all orders) from 13 clinicians. Outside the Memory Clinic, 356 tests were ordered by 91 clinicians: 145 orders (10.9%) from 33 neurology providers, 136 (10.2%) from 43 internal medicine providers, and 73 (5.5%) from 15 family medicine providers (one additional otolaryngologist ordered two tests). Although Memory Clinic clinicians generated most orders, over half of ordering clinicians (n = 58; 56%) were PCPs, reflecting broad early adoption across the health system. These early volume metrics describe adoption rather than effectiveness and should be interpreted as signals of feasibility rather than evidence of improved access or efficiency

### 1.3 Cognitive assessment visit (CAV) with ehr‐embedded decision support

To equip PCPs with a practical, standardized approach to dementia evaluation, we developed the Cognitive Assessment Visit (CAV), an EHR‐integrated workflow aligned with Center for Medicare and Medicaid Services requirements for Cognitive Assessment and Care Plan Service billing, Current Procedural Terminology (CPT) Code 99483. This alignment ensures comprehensive, clinically rigorous evaluations while encouraging adoption through sustainable reimbursement. The CAV was iteratively designed by a multidisciplinary team of PCPs and neurologists and refined through ongoing feedback.^21^ Because our immediate gap is inconsistent handling of memory complaints in primary care, the CAV is designed to support PCPs when cognitive concerns are identified during routine care (i.e., patient/family report or with screening during a Medicare Annual Wellness Visit). We plan to add population‐level EHR risk identification, but only after these PCP workflows are stable and scalable.

The CAV embeds structured evaluation, decision support, and automated documentation to reduce practice variability, strengthen PCP confidence, and align with reimbursement. The workflow includes a semi‐structured clinical history template, validated cognitive screening tools,^22^, ^23^, ^24^ preconfigured order sets for labs and biomarkers, EHR‐based prompts that guide diagnosis, biomarker use, referrals, and automated generation of a clinical note aligned with CPT 99483 reporting requirements.

#### 1.3.1 Initial uptake

In the first four months, 59 CAVs were completed by nine PCPs in Family Medicine, where training was initially targeted. Early use demonstrated that structured evaluations can generate documentation that supports billing under CPT 99483. Clinicians were encouraged to schedule 40‐ or 60‐minute appointment blocks for the CAV, with the enhanced reimbursement helping justify the extended appointment time. In some cases, PCPs completed the CAV within a standard 20‐minute visit, particularly when long‐standing patient relationships allow more efficient assessments. Some health system clinics are self‐organizing around the CAV in flexible ways, for example, by having trained nurses performed testing in advance, or having designated clinicians pre‐scheduled for performing CAVs and providing consultative feedback for their referring colleagues.

Between June and September 2025, the number of patients with documented MCI diagnoses seen by Family Medicine clinicians was 30% higher than the same period in 2024 (from 349 to 460) despite a stable at‐risk (age 65+) population. Additionally, this growth in MCI diagnoses was not associated with increased referrals from Family Medicine to the Memory Clinic (22 in 2025 vs 26 in 2024). In the first six months following ‐based biomarker implementation, at least 12 patients were referred from Family Medicine directly to the Anti‐Amyloid Treatment Clinic (vs 1 in 2024; outlined below). Although these observations are preliminary and descriptive, they suggest potential changes in diagnosis and referral patterns that warrant future evaluation.

Despite these apparent gains, time constraints remain a barrier. PCPs report difficulty integrating the CAV within routine schedules, prompting interest in streamlined tools such as a brief, EHR‐integrated “SmartPhrase” text shortcut to capture key cognitive and functional elements for triage and referral to the Anti‐Amyloid Treatment Clinic. To further improve efficiency, the BHCA is piloting an accelerated CAV model incorporating digital cognitive testing to increase efficiency in the clinic and enable timely, high‐quality evaluations within primary care, as others have done.^19^

### 1.4 Multidisciplinary co‐management memory clinics

Memory clinics face access challenges due to limited specialist capacity. Comprehensive evaluations remain the gold standard but are resource‐intensive and not always necessary at entry. Modern memory clinics must now serve multiple purposes – complex diagnosis, longitudinal support, and management of new pathology‐modifying therapies – but most still rely on a single pathway that may not efficiently serve patients with specific needs.

To address this, we reorganized the KU Memory Care Clinic into purpose‐built sub‐clinics aligned with patient needs and supported by APPs and social workers. This tiered model allows APPs to support PCPs in co‐managing straightforward cases, preserves cognitive neurologist time for complex presentations, and enables more timely evaluation for those eligible for early treatment options.

- *Anti‐Amyloid Treatment Clinic (APP‐led, Physician oversight)*: APPs coordinate the evaluation to determine eligibility, lead the shared decision‐making process, initiate therapy, and monitor for amyloid‐related imaging abnormalities (ARIA). Since launch, > 500 patients have been evaluated and > 275 have been prescribed one of the anti‐amyloid therapies. Therapy initiation is approved by neurologists, typically through completion of an e‐consult.^25^
- *Comprehensive Memory Support Clinic (APP and Social Work with Physician partners)*: Provides longitudinal management, caregiver training, behavioral management, safety planning, and care coordination. While not formally labeled as palliative care, this clinic delivers longitudinal, quality‐of‐life–focused support across the disease course through a co‐management model, linking families to Cognitive Care Network social workers for ongoing counseling, education, and support.
- *Complex Diagnostic Clinic (Neurologist‐led, APP‐supported)*: Serves as the traditional model for atypical or complex cases. Patients first complete an intake with an APP, who orders an appropriate workup (e.g., MRI, blood biomarkers) and may redirect to other sub‐clinics when appropriate. Intake visits include a 35‐minute embedded neuropsychological battery to accelerate the diagnostic process.
- *Memory Care e‐Consults (Neurologist‐led)*: After completing a CAV, PCPs can request an e‐consult from cognitive neurology, a chart‐based electronic review providing rapid specialist guidance without an in‐person visit. This pathway supports PCP confidence, reduces unnecessary referrals, and helps triage patients to appropriate sub‐clinics.

#### 1.4.1 Early impact of memory clinic reorganization

Since reorganization, clinic throughput has increased by more than 60% over two years (4,630 visits in FY2023 to 6,527 in FY2025, with FY2026 projected to exceed 7,700) with only the addition of an APP in 2024. More than 330 e‐consults have been completed during this period. These changes have improved patient flow and reduced wait times. Referrals to the Anti‐Amyloid Treatment Clinic are typically scheduled within 1‐2 months, while those to the Complex Diagnostic and Support Clinics now average about 3 months, down from 6‐12 months several years ago. Despite these gains, throughput remains a challenge, and we continue to refine triage procedures and work to expand clinical capacity. With approximately 250‐300 new referrals each month, only about one‐third are scheduled based on a triage process that requires documentation of cognitive change and objective testing (bedside or formal). Newly developed visit types and referral orders in the EHR further align scheduling with sub‐clinic structure and clarify options for referring PCPs. This new structure provides a flexible foundation to continue evolving more efficient operations.

### 1.5 Enabling and sustaining change

To transform dementia care in primary care, new diagnostics must be paired with systems that support practical and sustainable use. At our health system, BHCA was feasible because it leveraged legacy system enablers developed through the NIH‐funded ADRC. Additional implementation strategies were deliberately developed in partnership with primary care providers to expand access and adoption. Importantly, BHCA did not launch as a centrally designed program built from scratch; it evolved iteratively as a pragmatic response to demand and workflow constraints. Below, we describe the implementation strategies and system enablers that supported early adoption and are central to sustaining the effort. Table 2 outlines our current strategies for implementation and sustainining activity.

**TABLE 2:** Implementation strategies and system enablers.

Focus area	Key elements
**Implementation Strategies**	
PCP Training	CME‐accredited videos and training sessions
Financial & Workflow Incentives	CPT 99483 reimbursement for CAV; streamlined referral pathways with rapid feedback.
Clinical Champions	Physician and APP leaders modeling use and providing peer support
Workflow Refinement	Ongoing EHR usability testing and structured clinician feedback cycles
**System Enablers and Supporting Programs**	
Cognitive Care Network (CCN)	Social work–led care model providing patient/family support and professional training
APP Dementia Fellowship	6‐month paid dementia care training program for APPs
Neuropsychology Workflows	Streamlined 35‐minute intake and 25‐minute treatment batteries improve throughput and reduce delays for therapy eligibility.
Pathology & Laboratory Medicine	CAP/CLIA‐certified infrastructure for rapid adoption of new diagnostics in collaboration with the KU ADRC Biomarker Core
Lifestyle & Caregiver Education	Embeds prevention and caregiver training via LEAP! and MyAlliance programs.
Brain Health Biobank	Collects routine clinical, imaging, and biospecimen data to advance research

#### 1.5.1 Implementation strategies

To enhance adoption, scalability, and sustainability, BHCA components are supported by strategies developed in response to PCP‐identified barriers. These efforts include CME‐accredited training modules for biomarkers (15‐minute online), diagnosis and treatment (90‐minute online), and a full‐day live program, with 12 PCPs formally trained in Family Medicine and expansion underway across departments.^26^, ^27^ Clinicians who order their first biomarker receive a personalized email with links to training resources. Financial and workflow incentives, such as CPT 99483 reimbursement, and streamlined referral pathways, are intended to offset productivity concerns. Physicians and APP champions embedded in the clinics are meant to model use, troubleshoot barriers, and mentor peers, while ongoing EHR usability testing and feedback cycles emphasize continual refinement for efficiency and practicality.

#### 1.5.2 System enablers

The BHCA also builds on longstanding programs that expand workforce capacity, provide patient and caregiver support, and connect clinical care with discovery science. For example, the KU ADRC launched a 6‐month paid *APP Dementia Fellowship* in 2023 to train APPs in dementia care, with a goal of rapidly expanding capacity.^28^ The *Cognitive Care Network (CCN)* is a dedicated social work team that supports patients and families after a dementia diagnosis through education, counseling, and resource navigation.^29^ In FY2025, the team supported 1,881 families referred from the Memory Clinic. The CCN is also working across the health system's broader social work programs with social work training initiatives to expand access to high‐quality post‐diagnostic support,^30^ and has trained more than 50 practicing social workers and over 100 students in dementia‐specific care in the past year. The CCN provides a key outlet for post‐diagnostic support that PCPs have traditionally expressed concerns about.^26^ *Education and engagement programs* (LEAP!^31^ and MyAlliance for Brain Health^29^) embed brain health and caregiver training into CAV workflows that emphasize positive health behaviors for the maintenance of independence, comorbidity reduction, and psychosocial support. The *Neuropsychology Service* redesigned workflows with a 35‐minute intake battery integrated into the Memory Clinic and a 25‐minute rapid assessment to support treatment decision, improving throughput while maintaining diagnostic rigor.^25^ The *Department of Pathology and Laboratory Medicine* provides CAP/CLIA‐certified infrastructure that interfaced with the KU ADRC Biomarker Core to enable rapid validation and integration of new biomarker assays into clinical care.

## 2 DISCUSSION

Our experience provides early proof of concept that a multi‐tiered, systems‐level approach can expand capacity for dementia diagnosis and management within our health system. The BHCA model is designed to increase diagnostic rates, shift identification to earlier disease stages, and improve accuracy while reducing reliance on cognitive neurologists. We have observed rapid uptake of plasma biomarker testing, early adoption of the CAV workflow, and encouraging provider feedback supporting this integrated approach. These early results establish a foundation for change, although continued refinement will be essential as new tools and therapies emerge.

AD biomarkers are a major advance, but they will not transform dementia diagnosis on their own. Their impact depends on integrating them with tools, workflows, and clinical supports that will guide appropriate use, define the clinical context for accurate interpretation, and provide support after diagnosis. Biomarkers create the opportunity to detect disease earlier, but to be optimally used they must be paired with interventions that equip and motivate PCPs to act, answering the “now what” questions of how to initiate anti‐amyloid therapy, connect patients with caregiver education, manage behavioral symptoms, and coordinate supportive or palliative care within a collaborative system. Sustainably integrating these tools into practice requires aligning with system‐level strategies that are mutually reinforcing.

Traditional memory clinics often operate as one‐size‐fits‐all models, directing all patients through the same pathway regardless of complexity and creating bottlenecks by over‐relying on cognitive neurologists. These clinics must now serve multiple roles – complex diagnosis, therapy initiation, and long‐term support – but are rarely structured to match patients with the appropriate purpose of care. We reorganized the KU Memory Care Clinic into a multi‐tiered model with distinct sub‐clinics to align resources with patient needs while expanding throughput with APP and social work integration. This team‐based structure parallels chronic disease care models that improved outcomes through standardized workflows and shared roles,^14^, ^15^, ^16^ illustrating how dementia care can evolve toward a new model of co‐management that empowers primary care while preserving specialist expertise for complex cases and advanced therapies.

Broad adoption of new care models will also require aligning with reimbursement and policy incentives. The CMS GUIDE Model represents an important policy and payment development supporting longitudinal dementia care, caregiver support, and care navigation. The BHCA was designed primarily to address diagnostic and therapeutic capacity and therefore complements, rather than replaces, GUIDE‐aligned services. Several BHCA components, including dementia care navigation and caregiver support through the CCN and structured care planning workflows, align with GUIDE priorities. However, participation in GUIDE and payment‐model–specific financial analyses were beyond the scope of this early implementation report. At present, there is limited transparency or understanding of sustainable reimbursement and financing pathways, which creates uncertainty and slows investment. Policy levers such as CPT 99483 can incentivize comprehensive assessments and workforce programs, such as APP dementia fellowships^28^ and dementia‐focused social work training,^30^ can expand capacity. Health systems will need to invest in infrastructure, but payers and policymakers must create the conditions that make such investments viable.

Our experience has limitations. The primary limitation of this early implementation phase is the absence of downstream outcome measures such as changes in time‐to‐diagnosis, wait times for specialty evaluation, or patient‐ and caregiver‐reported outcomes, which are the focus of our ongoing evaluation. The data are early and reflect implementation within an extensive academic health system with substantial infrastructure, which limits generalizability. Biomarker testing was provided at no cost to patients through support from Fujirebio Diagnostics through the Davos Alzheimer's Collaborative Healthcare System Preparedness Accurate Diagnosis program,^19^ making adoption unusually frictionless; out‐of‐pocket costs and insurance coverage will likely shape future use. The appropriate use of biomarkers across > 1,300 in‐house p‐tau217 orders is still being evaluated to ensure clinically meaningful practice. Among completed tests, 73.6% in the Memory Care Clinic and 63.1% outside were “elevated,” suggesting appropriate use across settings, though further evaluation is ongoing. Adoption and sustainability in resource‐limited settings remain untested, and long‐term outcomes such as diagnostic accuracy, timeliness of care, and impact on patient and caregiver experience are not yet known.

The BHCA is an early, pragmatic case example of how one health system responded to rising dementia prevalence and an evolving diagnostic/therapeutic landscape by embedding biomarkers and structured evaluation workflows into primary care and strengthening co‐management with memory specialists. While this report focuses on feasibility and early adoption rather than definitive impact, we view the same PCP‐facing tools and workflows as a foundation for broader dissemination over time. As the BHCA matures, evaluation will focus on outcomes not yet assessable during early implementation, including time to diagnosis, patient and caregiver experience, clinician workload, and cost of care from health system and payer perspectives; these analyses will require longer follow‐up, appropriate comparators, and a more stable operational state.

## CONFLICT OF INTEREST STATEMENT

J.M.B. reports institutional clinical trial support from Eli Lilly, Amylyx, Biogen, Eisai, AbbVie, Roche, Ionis, and AstraZeneca; consulting fees from Renew Research, Eisai, Eli Lilly, Labcorp, Roche, AstraZeneca, Renew Biotechnologies, AbbVie, and Novo Nordisk; honoraria and travel support from Eli Lilly; and advisory board participation for Intra‐Cellular Therapies, Inc. R.A.T. reports institutional clinical trial support from Eisai, ONO, Novartis, and Eli Lilly, honoraria from the Alzheimer's Association of Western Kansas, and travel reimbursement from the Alzheimer's Therapeutic Research Institute. M.B.N. reports consulting fees, honoraria, and travel support from the American Society of Clinical Pathology, the Association for Diagnostics and Laboratory Medicine, and Lighthouse Lab Services. A.A. author reports personal consulting fees from CognitionMetrics LLC. All remaining authors report no competing financial or non‐financial real or perceived interests beyond institutional grant support. Author disclosures are available in the supporting information.

## Supporting information



Supporting Information
